# Multiple Uric Acid Bladder Stones: Clinical Presentation and Endoscopic Management

**DOI:** 10.1089/cren.2016.0134

**Published:** 2017-02-01

**Authors:** Fabio Cesar Miranda Torricelli, Shih-Chieh Jeff Chueh, Shujane Shen, Manoj Monga

**Affiliations:** ^1^ University of Sao Paulo Medical School, Sao Paulo, Brazil.; ^2^ Cleveland Clinic Glickman Urological and Kidney Institute, Cleveland, Ohio.

**Keywords:** endoscopy, metabolic stone, obstruction, uric acid, urolithiasis

## Abstract

***Background:*** Bladder urinary calculi occur in 3%–8% of men with bladder outlet obstruction, and although most of them are composed of calcium, in a few cases uric acid bladder stones are diagnosed.

***Case Presentation:*** We present clinical images and therapeutic management of a 65-year-old diabetic man with significant prostate enlargement and >30 bladder stones, the largest being 17 mm. Despite the large stone burden, the patient was managed by cystolithotripsy. Remarkably, stone composition analysis revealed 100% uric acid stone. Intraoperative and postoperative course were uneventfully.

***Conclusion:*** Uric acid bladder stone pathogenesis seems to be multifactorial with local and systemic factors contributing in different manners and even large stone burdens may be cystoscopically managed.

## Introduction and Background

Bladder urinary calculi are usually secondary to bladder outlet obstruction (BOO) in men, and although lower urinary tract symptoms are commonly associated with BOO, bladder stones develop only in 3%–8% of cases.^1^ The pathophysiology is not completely understood, but most bladder stones are composed of calcium.^1^ In a few cases, uric acid bladder stones were reported, commonly associated with systemic and local factors.^2^

## Presentation of Case

**FIG. 1.**
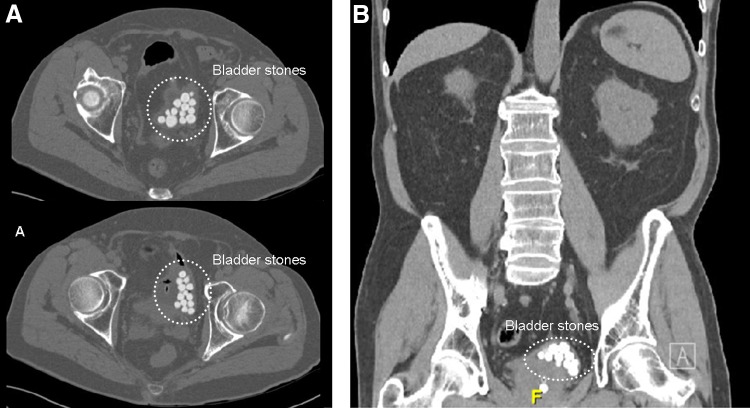
Computed tomography scan showing multiple uric acid bladder stones. **(A)** Axial view; **(B)** coronal view.

**FIG. 2.**
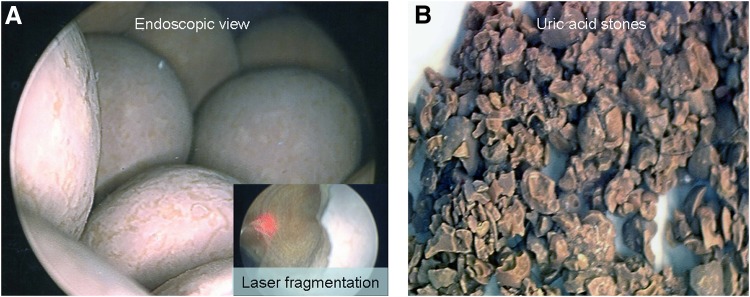
**(A)** Endoscopic view and laser cystolithotripsy; **(B)** uric acid stone fragments after lithotripsy.

We present clinical images and therapeutic management of a 65-year-old diabetic man who presented with acute urinary retention and gross hematuria in our emergency department. He had reported lower urinary tract symptoms for several years, with good symptomatic response to alpha-blockers. After Foley catheter placement, computed tomography scan was obtained to evaluate for hematuria, revealing significant prostate enlargement and >30 bladder stones, the largest being 17 mm (Fig. 1). Bladder stones had 700 Hounsfield units. Despite the large stone burden, the patient underwent transurethral cystolithotripsy with holmium laser. Bladder was in good conditions, presenting few trabeculations. A 1000μm laser fiber was utilized through a 21F rigid cystoscope with settings of 1.0 J and 10 Hz at the beginning and 1.5 J and 12 Hz at the end of the procedure. Fragmentation of all the stones was effective (Fig. 2). Fragments were actively extracted with Ellik evacuator. Transurethral resection of the prostate (TURP) was not performed at the same time of cystolithotripsy because the patient did not present important voiding complaints.

The Foley catheter was removed on the fourth postoperative day. There were no intraoperative or postoperative complications. Stone composition analysis revealed 100% uric acid stone. Patient's metabolic evaluation revealed a urinary pH of 5.0. Then, potassium citrate was prescribed.

## Discussion and Literature Review

The pathogenesis of uric acid bladder calculi has not been established. Li et al. in a study of 77 patients with uric acid urolithiasis (55 in the kidney and 22 in the bladder) reported that local factors were more important than systemic factors in the formation of uric acid bladder stone, because they did not find any significant difference in the urine stone risk analysis between patients with kidney and bladder stones.^2^ However, Childs and colleagues. compared men with and without bladder calculi who underwent surgical intervention for benign prostatic hyperplasia and reported that patients with bladder stones had lower 24-hour urine pH (median 5.9 *vs* 6.4; *p* = 0.02), lower 24-hour urinary magnesium (median 106 *vs* 167 mmol; *p* = 0.01), and increased 24-hour urinary uric acid supersaturation (median 2.2 *vs* 0.6; *p* < 0.01). In this study, most of the patients had calcium bladder stones (84%), and no correlation between stone composition and urinary pH was found, but all patients with uric acid stones had a 24-hour urine pH of less than 5.8, suggesting a metabolic contribution to stone formation.^1^ In our case, local (BOO) and systemic (low urinary pH) factors contributed to uric acid stone bladder formation. Furthermore, our patient had severe diabetes, which is also related to uric acid stone formation.

Regarding the bladder stone treatment, the stone size is the most important parameter when choosing between open and endoscopic techniques. Cystolithotripsy is usually reserved to stone burden lower than 2–3 cm, but in our case it was feasible and safe. Although we opted for transurethral laser lithotripsy, these stones could have also been managed by transurethral or percutaneous ultrasonic and/or pneumatic lithotripsy. Percutaneous approaches allow for improved drainage and straightforward removal of stones/fragments up to 1.0 cm (assuming a 30F sheath).

Controversy exists as to whether a bladder outlet procedure (e.g. TURP) should be performed in a staged or simultaneous setting.^3,4^ Philippou et al. prospectively compared 32 patients with bladder calculi who underwent TURP during the same session with 32 patients who underwent medical therapy for benign prostatic hyperplasia (tamsulosin plus finasteride). In this study, both groups experienced statistically significant postoperative improvements in the International Prostate Symptom Score (IPSS), peak urinary flow rate, and postvoid residual urine volume; however, patients who underwent TURP experienced a more pronounced improvement in the IPSS (*p* = 0.02) and peak urinary flow rate (*p* = 0.001). Furthermore, 11 (34.3%) patients initially managed by medications needed TURP during follow-up with medical management considered to have failed.^3^ In our case, the patient is still under medication and presents no voiding complaints.

Patient is taking potassium citrate to increase urinary pH and prevent stone recurrence. Furthermore, he is still on alpha-blocker therapy to facilitate bladder emptying.

## Conclusion

Uric acid bladder stone pathogenesis seems to be multifactorial with local and systemic factors contributing in different manners and even large stone burdens may be cystoscopically managed.

## Abbreviations Used

BOO: bladder outlet obstruction
TURP: transurethral resection of the prostate
IPSS: International Prostate Symptom Score
